# Ultrasound-guided Placement of Single-lumen Peripheral Intravenous Catheters in the Internal Jugular Vein

**DOI:** 10.5811/westjem.2018.6.37883

**Published:** 2018-07-26

**Authors:** Tony Zitek, Elizabeth Busby, Heather Hudson, John D. McCourt, Jamie Baydoun, David E. Slattery

**Affiliations:** * University of Nevada, Las Vegas School of Medicine, Department of Emergency Medicine, Las Vegas, Nevada; † University Medical Center of Southern Nevada, Department of Emergency Medicine, Las Vegas, Nevada; ‡ Kendall Regional Medical Center, Department of Emergency Medicine, Miami, Florida

## Abstract

**Introduction:**

The peripheral internal jugular (IJ), also called the “easy IJ,” is an alternative to peripheral venous access reserved for patients with difficult intravenous (IV) access. The procedure involves placing a single-lumen catheter in the IJ vein under ultrasound (US) guidance. As this technique is relatively new, the details regarding the ease of the procedure, how exactly it should be performed, and the safety of the procedure are uncertain. Our primary objective was to determine the success rate for peripheral IJ placement. Secondarily, we evaluated the time needed to complete the procedure and assessed for complications.

**Methods:**

This was a prospective, single-center study of US-guided peripheral IJ placement using a 2.5-inch, 18-gauge catheter on a convenience sample of patients with at least two unsuccessful attempts at peripheral IV placement by nursing staff. Peripheral IJ lines were placed by emergency medicine (EM) attending physicians and EM residents who had completed at least five IJ central lines. All physicians who placed lines for the study watched a 15-minute lecture about peripheral IJ technique. A research assistant monitored each line to assess for complications until the patient was discharged.

**Results:**

We successfully placed a peripheral IJ in 34 of 35 enrolled patients (97.1%). The median number of attempts required for successful cannulation was one (interquartile range (IQR): 1 to 2). The median time to successful line placement was 3 minutes and 6 seconds (IQR: 59 seconds to 4 minutes and 14 seconds). Two lines failed after placement, and one of the 34 successfully placed peripheral IJ lines (2.9%) had a complication – a local hematoma. There were, however, no arterial punctures or pneumothoraces. Although only eight of 34 lines were placed using sterile attire, there were no line infections.

**Conclusion:**

Our research adds to the growing body of evidence supporting US-guided peripheral internal jugular access as a safe and convenient procedure alternative for patients who have difficult IV access.

## INTRODUCTION

When patients with difficult intravenous (IV) access present to the emergency department (ED), they may experience significant delays in care.^1^ A recently described technique – the peripheral internal jugular (IJ) or “easy IJ” – provides a novel means to establish IV access on these patients. This technique, first described in 2009, involves placement of a peripheral IV catheter in the IJ vein under ultrasound (US) guidance.^2^ Subsequently, several small studies have concluded that this is a fast and safe procedure.^3^–^8^ Moreover, a recent review article calculated that the literature has reported 154 patients in whom peripheral IJs have been attempted, and it concluded that peripheral IJs are fast, effective, and have low complication rates. However, it also concluded that further data are needed.^9^

With the above-mentioned studies as support, several physicians in our hospital have begun placing peripheral IJs; however, a number of other physicians, nurses, administrators, and support staff have questioned the safety of placing a central line without following all the typical precautions associated with an IJ central line (full sterile barrier precautions, BIOPATCH® placement, post-procedure chest radiograph, etc.). Indeed, it may be argued that a peripheral IJ is a central line as the Centers for Disease Control and Prevention defines it – “an intravascular catheter that terminates at or close to the heart or in one of the great vessels” – and clarifies that the type of device inserted does not determine if the line qualifies as a central line. 10

Therefore, as a relatively new technique, a number of details regarding how peripheral IJs should be placed and the safety of the procedure are uncertain. Thus, we believe it is important to add to the existing literature more information about the speed and, especially, the safety of US-guided IJ vein peripheral cannulation. We performed a prospective evaluation of peripheral IJ placement on a convenience sample of ED patients who required IV access and had difficult IV access.

### Study Aims and Objectives

Our primary outcome was to determine the rate at which attempted peripheral IJs are successful in a heterogeneous group of operators. Secondarily, we sought to determine mean time to successful line placement and the frequency of complications.

## METHODS

This was a prospective case series at a single, urban, academic emergency department (ED) with an annual census of about 77,000. We evaluated the placement of peripheral IJs on a convenience sample of adult ED patients with difficult IV access who required IV access for medical management. Our hospital’s institutional review board (IRB) approved this study, and we registered it on clinicaltrials.gov (NCT03231345). All patients on whom a peripheral IJ was attempted signed written, informed consents.

Inclusion criteria were at least two unsuccessful attempts at peripheral IV access by ED nursing staff and age >18. We excluded patients if they were critically ill with clinical indications for emergent triple-lumen catheter access, had an overlying skin infection, had an external jugular vein that was easily visible for cannulation, were in law enforcement custody, were pregnant, or were unable to give consent. Emergency medicine (EM) residents who had placed at least five central lines in the IJ vein were eligible to place peripheral IJs for this study after watching a 15-minute lecture about the technique. Five EM attending physicians who had previous experience placing peripheral IJs were also eligible to place the lines for this study.

**Population Health Research Capsule**

What do we already know about this issue?

The peripheral internal jugular (IJ) is an alternative means of obtaining vascular access in patients with difficult vascular access, but some details regarding the procedure and its safety are uncertain.

What was the research question?

Can a heterogeneous group of emergency physicians with minimal training safely and efficiently place peripheral IJ lines?

What was the major finding of the study?

Peripheral IJs were successfully and rapidly placed on 34 of 35 patients with only one complication – a local hematoma.

How does this improve population health?

This study provides additional data that peripheral IJs are a reasonable option for patients with difficult vascular access.

The technique for peripheral IJ placement for this study was as follows. The skin was prepped with an alcohol swab or chlorhexidine. Direct US guidance with a linear transducer was required, and a sterile probe cover was recommended. Gloves were required, but sterile gloves were not mandated. The catheter used for the study was the Introcan Safety® catheter (Braun, Kronberg, Germany), a single-lumen 18-gauge, 2.5-inch catheter. Standard catheter-over-needle method was used, and the catheter was secured in typical fashion as for a standard IV start. We also requested that all providers order a chest radiograph (CXR) after line placement to rule out pneumothorax.

As described in more detail in the discussion section below, about halfway through enrollment, although no line infections had occurred, our institution’s patient safety committee mandated that we place peripheral IJs as if they were central lines with sterile technique, using full sterile barrier precautions, a sterile dressing, and a BIOPATCH®. Thus, there was an abrupt change in the means by which peripheral IJs were placed during the course of study. This change occurred despite prior IRB approval of a protocol that did not require sterile technique.

After consent, a trained observer watched the physician place the peripheral IJ, and the observer filled out a standard data collection form. The data collection form included location of the attempt (left or right IJ), the level of training of the physician placing the line, the equipment used, number of attempts, time to successful placement, post-procedure portable CXR results, immediate complications (arterial puncture, neck hematoma, or pneumothorax), equipment used, time to discontinuation of catheter, reason for catheter removal, and delayed complications (thrombus or line infection). Basic demographic information including age, gender, race, and body mass index were also recorded.

The number of “attempts” was defined as the number of times the needle punctured the skin. The time to successful placement began when the US probe touched the patient’s skin, and the time stopped when either blood was successfully withdrawn from the line or when the line was successfully flushed. A “line failure” occurred when a line that was initially successfully placed could no longer draw blood or be flushed. For patients who ended up getting admitted, a research assistant checked on the line once per day until the patient was discharged from the hospital. If the line had any problems or was discontinued, the research assistant would determine what the problem was or why it was removed. Two weeks after patients were discharged, a research assistant reviewed the medical records to determine if there was a positive blood culture that may not have been known about at the time of discharge.

At the time this study was conceived, the largest study about peripheral IJs included just 33 patients,^6^ so our goal was to enroll 50 patients in an attempt to make this the largest peripheral IJ study to date.

The primary outcome was successful cannulation of the IJ with a peripheral venous catheter. Secondary outcomes included time to placement, number of attempts, and complications.

## RESULTS

We enrolled 35 patients between August 2016 and September 2017. We did not achieve our goal of 50 patients because enrollment dramatically decreased after our hospital mandated that peripheral IJs be placed using full sterile barrier precautions, and the study was stopped early. Table 1 shows the baseline characteristics of the 35 enrolled patients.

With regard to the primary outcome, a peripheral IJ was successfully placed in 34 of 35 enrolled patients (97.1%; 95% confidence interval [CI] [85.1–99.9]). On first attempt, the line was successfully placed in 22 of 35 patients (62.8%; 95% CI [44.9–78.5]). The median number of attempts was one (interquartile range [IQR]:1 to 2), and the mean number of attempts was 1.41 (95% CI [1.24–1.58]). The median time to successful cannulation was 3 minutes and 6 seconds (IQR: 59 seconds to 4 minutes and 14 seconds). Line failure occurred in two cases, and both occurred within one hour of line placement. In one of those cases, the line failure occurred because the line was dislodged due to cardiopulmonary resuscitation. The appendix lists the number of attempts and time it took for successful cannulation for each of the 35 enrolled patients.

Of the 35 peripheral IJs attempted, 25 (71.4%) were attempted by a resident, and 10 (28.6%) were attempted by an attending physician. The difference in first-attempt success rates for residents and attending physicians was not statistically significant: 60% (95% CI [38.7 to 78.9]) for residents and 70% (95% CI [34.8 to 93.3]) for attendings, but the median time to cannulation was shorter for attendings. Table 2 shows a more detailed breakdown of success rates by level of training.

We tracked the equipment used by physicians for the placement of peripheral IJs. Although the catheter that was supposed to be used for this study was a 2.5-inch, 18-gauge catheter, in one instance a 1.25-inch, 18-gauge catheter was used. This occurred on the second attempt on subject 34, and this was the only subject on whom a peripheral IJ was not successfully placed. In addition, there was significant variability in the equipment used for peripheral IJ placement, in part, because of physician preference and in part, because our hospital mandated sterile technique after the study had already started (as described further below). Table 3 outlines the equipment used by physicians in our study for peripheral IJ placement.

One of the 34 successfully placed peripheral IJ lines (2.9%; 95% CI [0.1–15.3]) had a complication: a small hematoma that resolved spontaneously without incident. There were no arterial punctures, pneumothoraces, or line thrombi. In 30 of 35 cases, the absence of pneumothorax was confirmed by a post-procedure CXR. Although we told providers to order a CXR after attempting a peripheral IJ, in five cases this was not done. Upon a review of the medical records, all five of those patients were discharged from the hospital without incident, and there was no indication that anyone of those five was suspected to have a pneumothorax. Although the provider only used sterile attire in eight of 34 successfully placed lines, there were no line infections or cases of bacteremia in any of the enrolled patients. Lines were left in for an average of 58 hours, with a maximum of 339 hours.

Through the course of the study, it came to our attention that some technicians in the radiology department and some radiologists in our hospital were concerned that it might not be safe to give IV contrast through peripheral IJ lines because extravasation could be particularly harmful. Therefore, although we did not prospectively assess for contrast extravasation, we found through retrospective analysis that 13 enrolled patients (37.1%) had an IV-contrast radiologic study including seven computed tomography (CT) with IV contrast, two magnetic resonance images with IV contrast, and three nuclear medicine studies. One CT angiogram of the chest was done. There were no instances of contrast extravasation, but of note, the CT angiogram of the chest was read as having “suboptimal opacification of the pulmonary arteries.”

## DISCUSSION

The results of this study are consistent with other recent literature,^3^–^8^ suggesting that a peripheral IJ can be placed on the majority of patients with only one attempt. Our study was unique in that residents with minimal training placed the majority of lines, and they had high success rates with this procedure. Our study also adds to the growing body of literature that suggests that peripheral IJs are safe. Although our study was not large enough to estimate the rate at which serious complications such as pneumothoraces or line infections occur after the placement of peripheral IJs, the fact that none occurred in our study or other observational studies examining this technique^3^–^8^ suggests that these complications are very rare.

Despite the data in favor of the use of peripheral IJs for patients with difficult IV access, our study also demonstrates some of the difficulties that providers may have when trying to use what will be an unfamiliar line to others in the hospital. In particular, our hospital’s patient safety committee expressed concerns that we were not using full sterile barrier precautions for the central lines we were placing. Consequently, our IRB requested that we suspend the study until our principal investigator could talk to the physician in charge of the patient safety committee. This resulted in a protocol change in which we mandated that our physicians place the lines using sterile gloves and “sterile technique.” Subsequently, enrollment dropped and the study was stopped before meeting our goal enrollment of 50 patients.

Regarding whether peripheral IJs should be considered central lines, we maintain that they should not. The infection rate noted in studies about US-guided IJ central lines is about 10%.^11^ In our study there were zero cases of suspected line infections even without consistent sterile barrier precautions and even with some lines staying in place for a number of days. In previous studies,^3^–^8^ there have also been no reported line infections. Thus, while more data are needed to definitively determine the infection rate, the infection rate of peripheral IJs seems to be very low.

As to whether or not a CXR should be ordered after a peripheral IJ attempt, we would also argue that a CXR may not necessarily be required. The authors of some prior studies argue that routine CXRs are not needed after US-guided IJ central lines because the rate of complications is exceedingly low.^12^ Moreover, in our study and in previous studies^3^–^8^ there have been zero reported pneumothoraces from a peripheral IJ. Overall, it is difficult to see why it would be necessary for a provider to treat a peripheral IJ like a central line with full sterile barrier precautions and a post-procedure CXR when peripheral IV catheters placed in the external jugular vein (which is immediately adjacent to the internal jugular vein) are treated like any other peripheral IV lines.

## LIMITATIONS

This study had several limitations to consider. First, the study size was small and limited by selection bias. Therefore, our data are best interpreted by looking at our data along with the results from other studies about peripheral IJs. Second, as described above, our hospital patient safety committee compelled us to change our protocol during the course of data collection. This resulted in a change in the technique used for the procedure from nonsterile to sterile. Ideally, the entire study would have been done with nonsterile attire to provide more evidence that sterile attire is unnecessary.

Although we were more diligent about assessing for complications than some of the previous peripheral IJ studies, our protocol for assessing for complications could have resulted in some missed complications, such as delayed presentations of bacteremia. Also, in five cases the provider who attempted the peripheral IJ did not order a CXR after the procedure. It is thus possible (but unlikely) that we missed a pneumothorax. Finally, this study had no comparison group. A randomized trial comparing peripheral IJs to other US-guided peripheral IVs would help elucidate when peripheral IJs should be used in patients with difficult IV access.

## CONCLUSION

This study adds to the growing body of literature that suggests that peripheral IJs are a fast, safe, and easy alternative means for establishing IV access on patients with difficult IV access.

## Supplementary Information



## Figures and Tables

**Table 1:** Baseline characteristics of patients who received peripheral internal jugular line placement.

Characteristic	Number
Gender	
Female	25 (71.4%)
Male	10 (28.6%)
Age, mean (SD)	48.2 (15.6)
BMI, median (IQR)	25.7 (22.2, 31.0)
Race	
Caucasian	14 (40%)
African American	14 (40%)
Hispanic	6 (17.1%)
Asian	1 (2.9%)
Side of catheterization	
Left/right	16/19
Disposition	
Admitted	29 (82.9%)
Discharged	5 (14.3%)
Eloped	1 (2.9%)

*SD*, standard deviation; *BMI*, body mass index; *IQR*, interquartile range.

**Table 2:** Success rates of peripheral internal jugular line placement stratified by level of emergency physician training.

Level of training	Peripheral IJs attempted	Successful, n (%)	Successful on first attempt, n (%)	Median time to cannulation
PGY1	1	1, (100%)	1, (100%)	186 seconds
PGY2	12	12, (100%)	8, (66.7%)	197 seconds
PGY3	12	11, (91.7%)	6, (50%)	224 seconds
Attending	10	10, (100%)	7, (70%)	64 seconds

*PGY*, postgraduate year; *IJ*, internal jugular.

**Table 3:** Equipment used for peripheral internal jugular line placement.

Equipment	Frequency of use
Gloves	
Sterile	9 (25.7%)
Nonsterile	26 (74.3%)
BIOPATCH®	7 (20.6%)
Probe cover	30 (85.7%)

## References

[1] Witting MD (2012). IV access difficulty: incidence and delays in an urban emergency department. J Emerg Med.

[2] Moayedi S (2009). Ultrasound-guided venous access with a single lumen catheter into the internal jugular vein. J Emerg Med.

[3] Teismann NA, Knight RS, Rehrer M (2013). The ultrasound-guided “peripheral IJ”: internal jugular vein catheterization using a standard intravenous catheter. J Emerg Med.

[4] Butterfield M, Abdelghani R, Mohamad M (2017). Using ultrasound-guided peripheral catheterization of the internal jugular vein in patients with difficult peripheral access. Am J Ther.

[5] Moayedi S, Witting M, Pirotte M (2016). Safety and efficacy of the “easy internal jugular (IJ): an approach to difficult intravenous access. J Emerg Med.

[6] Kiefer D, Keller SM, Weekes A (2016). Prospective evaluation of ultrasound-guided short catheter placement in internal jugular veins of difficult venous access patients. Am J Emerg Med.

[7] Zwank MD (2012). Ultrasound-guided catheter-over-needle internal jugular vein catheterization. Am J Emerg Med.

[8] Butterfield M, Abdelghani R, Mohamad M (2017). Using ultrasound-guided peripheral catheterization of the internal jugular vein in patients with difficult peripheral access. Am J Ther.

[9] Gotlieb M, Russell FM (2018). How safe is the ultrasonographically guided peripheral internal jugular line?. Ann Emerg Med.

[10] Centers for Disease Control and Prevention (CDC) (2018). Bloodstream Infection Event (Central Line-Associated Bloodstream Infection and Non-central Line Associated Bloodstream Infection).

[11] Karakitsos D, Labropoulos N, De Groot E (2006). Real-time ultrasound-guided catheterisation of the internal jugular vein: a prospective comparison with the landmark technique in critical care patients. Crit Care.

[12] Hourmozdi JJ, Markin A, Johnson B (2016). Routine chest radiography is not necessary after ultrasound-guided right internal jugular vein catheterization. Crit Care Med.

