# Supplementary material for: Ultrasound-guided Placement of Single-lumen Peripheral Intravenous Catheters in the Internal Jugular Vein
Source: West J Emerg Med. 2018 Jul 26;19(5):808–12. doi: 10.5811/westjem.2018.6.37883 (PMC6123094; doi:10.5811/westjem.2018.6.37883)
Supplement: Supplementary file 1 [file wjem-19-808-s001.docx]

| **Patient #** | **# of Attempts** | **Successful?** | **Time to Completion (hrs, min, sec)** |
| --- | --- | --- | --- |
| 1 | 2 | Yes | 0:09:36 |
| 2 | 2 | Yes | 0:04:09 |
| 3 | 1 | Yes | 0:01:00 |
| 4 | 1 | Yes | 0:01:25 |
| 5 | 1 | Yes | 0:01:07 |
| 6 | 1 | Yes | 0:02:30 |
| 7 | 1 | Yes | 0:02:06 |
| 8 | 1 | Yes | 0:01:20 |
| 9 | 2 | Yes | 0:04:18 |
| 10 | 2 | Yes | 0:03:13 |
| 11 | 2 | Yes | 0:05:33 |
| 12 | 1 | Yes | 0:07:13 |
| 13 | 1 | Yes | 0:00:22 |
| 14 | 1 | Yes | 0:03:06 |
| 15 | 1 | Yes | 0:03:32 |
| 16 | 1 | Yes | 0:00:47 |
| 17 | 2 | Yes | 0:07:31 |
| 18 | 1 | Yes | 0:02:23 |
| 19 | 2 | Yes | 0:11:32 |
| 20 | 2 | Yes | 0:04:00 |
| 21 | 1 | Yes | 0:05:40 |
| 22 | 1 | Yes | 0:03:20 |
| 23 | 1 | Yes | 0:06:40 |
| 24 | 1 | Yes | 0:00:24 |
| 25 | 1 | Yes | 0:00:35 |
| 26 | 1 | Yes | 0:00:44 |
| 27 | 2 | Yes | 0:05:29 |
| 28 | 1 | Yes | 0:00:49 |
| 29 | 1 | Yes | 0:00:32 |
| 30 | 1 | Yes | 0:00:58 |
| 31 | 2 | Yes | 0:04:07 |
| 32 | 2 | Yes | 0:02:14 |
| 33 | 1 | Yes | 0:00:53 |
| 34 | 2 | No | Unsuccesful |
| 35 | 2 | Yes | 0:03:59 |

**Appendix**: Number of attempts and time to completion for each peripheral IJ enrollment.
